# GOTermViewer: Visualization of Gene Ontology Enrichment in Multiple Differential Gene Expression Analyses

**DOI:** 10.1177/11779322241271550

**Published:** 2024-09-18

**Authors:** Milene Volpato, Mark Hull, Ian M Carr

**Affiliations:** School of Medicine, University of Leeds, Leeds, UK

**Keywords:** Gene ontology enrichment, RNA seq analysis, next-generation sequencing, differential gene expression analysis

## Abstract

Gene ontology phrases are a widely used set of hierarchical terms that describe the biological properties of genes. These terms are then used to annotate individual genes, making it possible to determine the likely physiological properties of groups of genes such as a list of differentially expressed genes. Consequently, their ability to predict changes in biological features and functions based on alterations in gene expression has made gene ontology terms popular in the wide range of bioinformatic fields, such as differential gene expression and evolutionary biology. However, while they make the analysis easier, it is seldom easy to convey the results in a readily understandable manner. A number of applications have been developed to visualize gene ontology (GO) term enrichment; however, these solutions tend to focus on the display of aggregated results from a single analysis, making them unsuitable for the analysis of a series of experiments such as a time course or response to different drug treatments. As multiple pair wise comparisons are becoming a common feature of RNA profiling experiments, the absence of a mechanism to easily compare them is a significant problem. Consequently, to overcome this obstacle, we have developed GOTermViewer, an application that displays GO term enrichment data as determined by GOstats such that changes in physiological response across a number of individual analyses across a time course or range of drug treatments can be visualized.

## Introduction

Gene ontology (GO) terms are a widely used and evolving set of phrases used to define a gene product (proteins and noncoding RNAs) concerning their biological functions. These are curated by the Gene Ontology Consortium^1,2^ with terms added and removed with evolving biological understanding. Similarly, the terms linked to a gene may change over time as new biological links are found or disproved, typically following experimental or in silico analysis. While the current usage of GO terms has its limitations, they can still be very useful when describing biological phenomena such as attempting to determine a cell’s physiological response to an environmental stimulus or genetic mutation.

The specificity of terms ranges from very general to highly specialized, allowing them to be grouped together such that a more general term is the parent of a number of more specialized but related terms, which in turn are the parents of even more specific terms. These parent–child relationships are then linked together to form 3 distinct domains: biological process, molecular function, and cellular compartment to form a directed acyclic graph structure. Since a term can have a number of parents, the final structure resembles a road map, meaning it is possible to find several different ways to move from one term to a more distantly related term.

The analysis of gene expression using expression microarrays and/or next-generation sequencing of RNA is routinely performed to identify changes in gene expression profiles between various cohorts of biological material. These data can then be used to identify differentially expressed genes (DEGs) using software such as DeSeq2^3^ or edgeR.^4^ However, simple lists of DEG can often be too large to easily describe the resultant changes in sample physiology. To resolve this, several applications such as DAVID,^5^ TopGO,^6^ and GOstats^7^ have been developed that link DEG to their GO terms and then determine if a GO term is linked to more or fewer genes in the dataset than expected, when compared to a reference set of genes, such as all the genes expressed in a sample or those present in the organism’s genome. However, as with lists of DEG, extensive lists of enriched GO terms can be difficult to interpret. Consequently, several applications have been developed to visualize GO term enrichment data such as AmiGO,^8^ GO-Figure!,^9^ Gonet,^10^ NaviGO,^11^ QuickGO,^12^ and REVIGO.^13^

These applications typically display the data for a single analysis as a bubble plot, a network graph of linked terms, or a hierarchical tree graph. Of these, hierarchical graphs most accurately reflect the relationships between individual GO terms as their structure tends to be a simplified version of the relationships in the GO terms’ directed acyclic graph as defined by the GO Consortium, with GO terms omitted if they are uninformative. These graphs typically do not aggregate data from similar GO terms and, since they have a fixed structure, are useful for answering specific, detailed questions between 2 different enrichment experiments. By comparison, bubble plots tend to be useful for the display of global GO term enrichment trends, due to the number of terms present in an analysis, this is often achieved by aggregating data for similar terms into clusters.

While the structure of hierarchical graphs is strongly influenced by the relationships between GO terms, the structure of the other types of display is dictated by the strength of the relationships between GO terms in the displayed data set. The strength of a relationship is represented by their semantic similarity score, which can be calculated in a number of ways. Resnik^14^ published one of the earliest scoring methods used by this type of application,^15^ and was subsequently refined by Lin.^16^ These methods first identify common ancestral terms of a pair of GO terms and then evaluate the ancestral GO term based on the frequency at which it and its child terms are present in the gene ontology annotation (GOA) of the EBI’s UniProt knowledgebase.^17^ Where 2 terms have multiple common ancestors, the score may reflect the best score of the common ancestors or their average score. While the Resnik/Lin scores reflect the structure of the GO term graph, other scoring systems have been developed that measure the physiological link between different terms for instance CAS^11,18^ and PAS^8^ use the frequency by which 2 terms are referenced in the same PubMed abstract to determine their similarity, whereas IAS^12,19^ uses the frequency by which 2 terms are linked to proteins know to interact with each other. While it is important that these scores are both up to date and the GO terms used in their creation match those used in the enrichment analysis to avoid erroneous scores,^9,20^ Reijnders^9^ suggested that this may not be true for many analyses performed using online websites.

Due to the large number of enriched GO terms identified by some enrichment analyses, many applications perform a term reduction step to simplify the final display. Initial GO terms may be omitted from the display if they are too general, for instance, REVIGO ignores terms that have a frequency greater than 5% in the GOA. The semantic similarity scores are then determined for all the pairs of GO terms which are used to aggregate GO terms into clusters, eg, GO-Figure! combines a pair of GO terms if they have a semantic similarity score over 0.7. Once the clusters have been created, they may undergo a final filtering step either directed by the user, selecting those with a *P* value below a preset cutoff or by ranking the GO terms and selecting the top ’n’ GO terms.

While a single enriched GO term has obvious attributes such as a name, frequency of occurrence in GOA, number of DEG associated with it and enrichment *P* value, aggregate terms do not. Consequently, applications that merge GO terms often use a decision tree to determine a representative GO term whose values are then used to describe the cluster. Typically, these decisions are based on the term’s *P* value, parent–child relationship, and level of specificity, with a cluster’s attributes derived from the constituent GO term with the lowest *P* value and/or lowest level of specificity with parent terms beating child terms.

When displaying a GO term, its *P* value is generally used to determine the GO term’s colour, while for bubble plots, its size may reflect the number of DEG linked to it or by the number of GO terms a cluster represents. Interestingly, while REVIGO clusters GO terms, it may still display each individual GO term in a cluster but only labels the GO term that is found to be representative of that cluster.

The location of a GO term in a bubble plot may be determined by the unmodified values of the term or by calculating its coordinates based on the attributes of all the enriched GO terms in the display. For instance, NaviGO allows the user to select which method is used. One option is to use 2 different, user selected, similarity scores for each GO term as the GO term’s x and y coordinates. While the other option creates a multidimensional matrix of the semantic similarity scores of each pair of GO terms which is then reduced to 2 dimensions to determine the x and y coordinates for each GO term. To do this NaviGO uses an ‘S’ implementation of the multidimensional scaling algorithm,^21,22^ while GO-Figure! performs a similar task using the SciKit-Learn^23^ dimension reduction function. These coordinates may then be modified to ensure that 2 clusters do not completely or partially overlap before they are used to plot each cluster. Consequently, the location of a GO term in an image drawn using a pair of semantic similarity scores for the x and y axes is constant across different enrichment analyses but varies between plots of different enrichment analyses or the same analysis displayed using different cut off values when a single semantic similarity score is used to determine a GO terms position. Whichever method is used, more similar GO terms tend to be located closer to each other than less similar GO terms.

The visualization of GO terms in a network graph has similarities to both hierarchical graphs and bubble plots in that like hierarchical graphs, GO terms tend not to be aggregated and the data point is of a fixed size, while like bubble plots, the arrangement of the data points is determined by the semantic similarity score of each pair of terms as well as their relationship to each other in the GO term hierarchy. Unlike bubble plots the parent-child (ancestor-descendant) relationships are shown as lines that link pairs of related GO terms. Therefore, related terms can be identified by their proximity to each other as well as the presence of a connecting line. However, as the number of terms in a network graph increases, the presence of these lines can make the graph more confusing. Network graphs are the most flexible display type and may be extended to include other types of data, for instance, GOnet network graphs also includes differentially expressed proteins linked to the GO terms in the display. Consequently, the location of a GO term (and protein) is dependent on both its relationships with other features, like a hierarchical graph and like a bubble plot on the attributes all the visualized GO terms and proteins.

The majority of programmes created to visualize GO term enrichment datasets are designed to represent single sets of analysis. Two exceptions are VLAD,^24^ which was able to show the relative enrichment between at least 2 analyses, visualized as a hierarchical graph and GO-Figure! which states that it was designed with the comparison of multiple datasets in mind; however, VLAD no longer appears to be available and neither the paper or linked GitLab hosting page indicate how to perform enrichment comparisons with GO-Figure!. Consequently, we have developed GOTermViewer, an application that allows the easy comparison of multiple enrichment analyses such that it is possible to observe the progressive change in physiology over a time course or range of treatment regimes.

## Materials and Methods

### GO term enrichment data

Next-generation sequencing data for the GEO project GSE237377^25^ were downloaded from the NCBI SRA archive and converted to fastq files. The sequence data were trimmed to remove adaptor sequences and low-quality base calls using Cutadapt.^26^ The trimmed data were then aligned to the mouse reference genome (mm39) with reference to its RefSeq gene annotation obtained from the UCSC table browser^27^ using the STAR aligner.^28^ Reads aligned to the RefSeq gene sequences were then counted using the R package Rsubread.^29^ DEG for the 4 pair wise analyses (Table 1) was determined using DeSeq2, with the lists DEG from each pair wise analysis compared to a list of genes expressed in the samples to identify over- and underenriched GO terms using the R package GOstats.^7^ The results of the GO term over- and underenrichment were combined to produce a single results file for each pairwise analysis.

**Table 1.** Description of the analyses used in Figure 2.

Analysis	Reference samples	Modified samples
1 mg AN1284 versus saline in wild-type mice	Wild-type mice were given saline	Wild-type mice given 1 mg of AN1284
1 mg AN1284 versus saline in NASH mice	NASH mice were given saline	NASH mice given 1 mg of AN1284
5 mg AN1284 versus saline in wild-type mice	Wild-type mice were given saline	Wild-type mice given 5 mg of AN1284
5 mg AN1284 versus saline in NASH mice	NASH mice were given saline	NASH mice given 5 mg of AN1284

## Results

### Methodology

**Figure 1.**
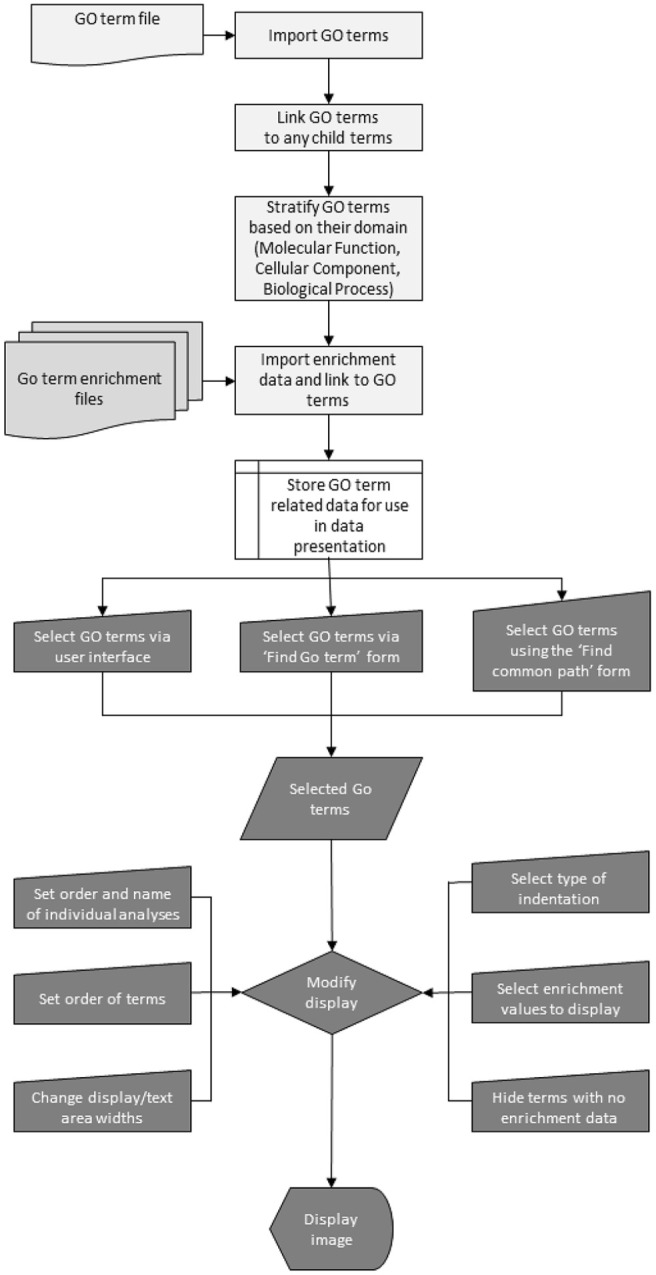
A high-level flowchart delineating the important stages of the data processing and visualization performed by GOTermViewer. The pale grey objects with black text identify tasks performed when importing the data (shown as the white object with black text), while the objects shaded in darker grey with white text indicate processes that modify the displayed data in response to user input.

The underlying steps involved in the processing and display of the data are outlined in Figure 1. The analysis consists of 2 distinct phases the importation of the GO terms and the linking of these GO terms to the enrichment data (shown as light grey objects with black text in Figure 1) and then the user-driven GO term selection and display (shown as dark grey objects with white text in Figure 1). Initially, the gene ontology file is processed with data for each GO term retained. Once all the terms have been imported, each term is linked to any child terms before being placed in 1 of the 3 domains ( ‘Molecular Function’, ‘Cellular Component’, and ‘Biological Process’). The current version of the GO terms contains ~47 000 GO terms with over 91 000 parent–child relationships, giving over third of a million unique paths across the directed acyclic graph from a root term to a term with no child terms of its own. Consequently, the terms are stored as 3 unstructured collections, one for each domain with any paths across the directed acyclic graph constructed on the fly when needed. While this significantly reduces the loading time and memory requires for storing the data, it dramatically increases the complexity of the programme such that it is no discernible lag when modifying the display in response to user input.

### Implementation

**Figure 2.**
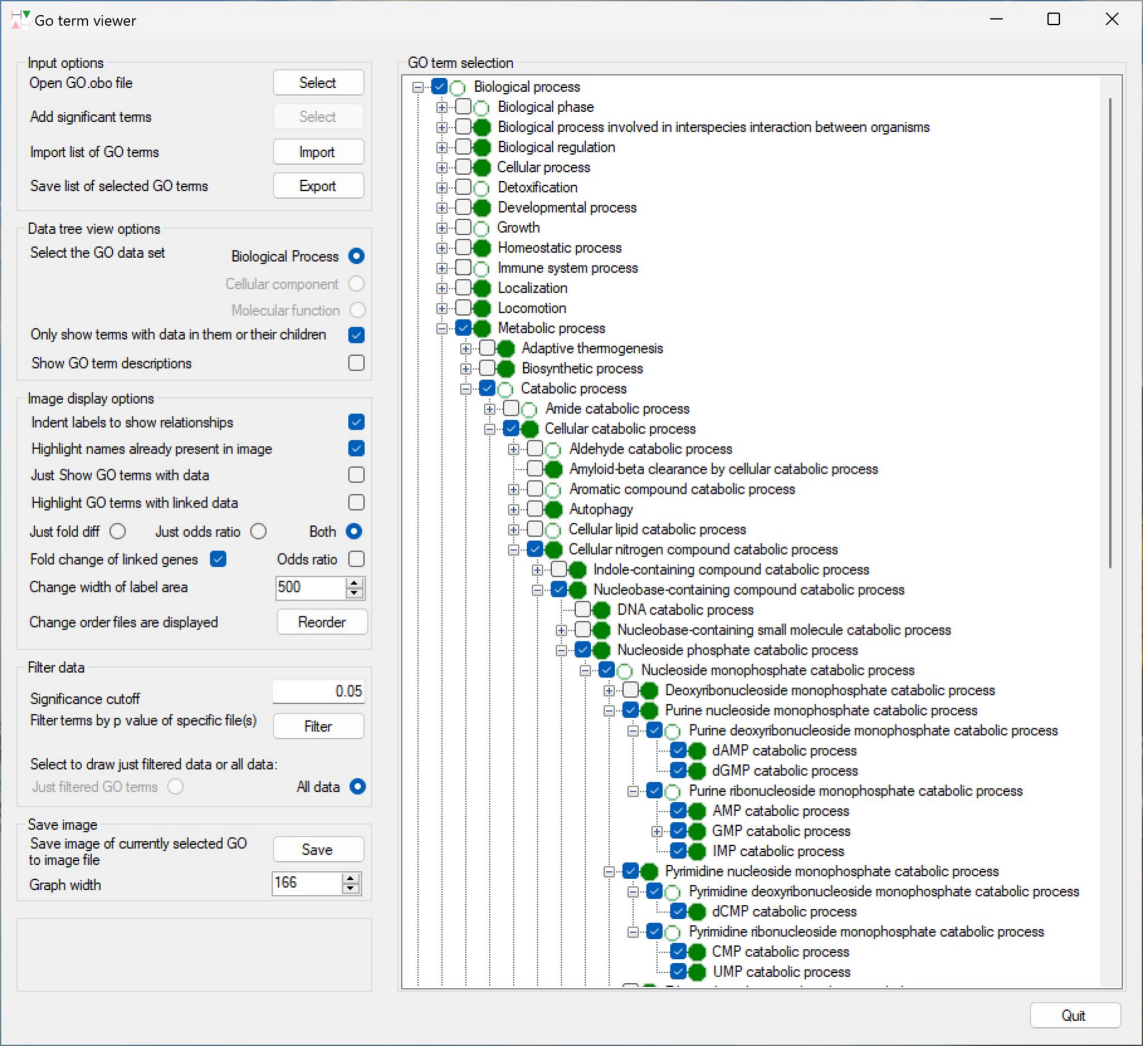
The primary window of GOTermViewer showing the display options to the left and a partially expanded tree of GO terms to the right.

GOTermViewer is a Windows desktop application written in C#, designed to visualize the results of GO term enrichment analyses from a series of related differentially expression experiments. The application principally consists of 2 windows, the primary window containing all the data display options which modify how the data are displayed on the secondary window. The right side of the primary window consists of a tree view panel, with each GO term represented as a node which can be expanded to show its child nodes. GO term nodes with enrichment data are shown with a green disc icon, while terms without data, but whose child terms do have data are identified with a green circular icon. By default, terms that on not linked to any data are hidden, but if displayed are identified by a pink disc. Selecting a node causes its enrichment data as well as that of any parent terms to be displayed in the secondary window (if required data from parent terms can be hidden) (Figure 2). To identify the location(s) of GO terms in the tree view, it is possible to search for either individual terms or the nearest common ancestor to a number of related GO terms. Since the tree view displays the GO terms as a set of all possible paths from the root GO term to each childless GO term, a term may occur numerous times in the tree. Consequently, it is advisable to consider which path is selected, for instance when viewing data for RNA catabolism it may prove to be more informative to select the path that passes through the Macromolecule metabolic process term rather than Cellular nitrogen compound catabolic process term.

**Figure 3.**
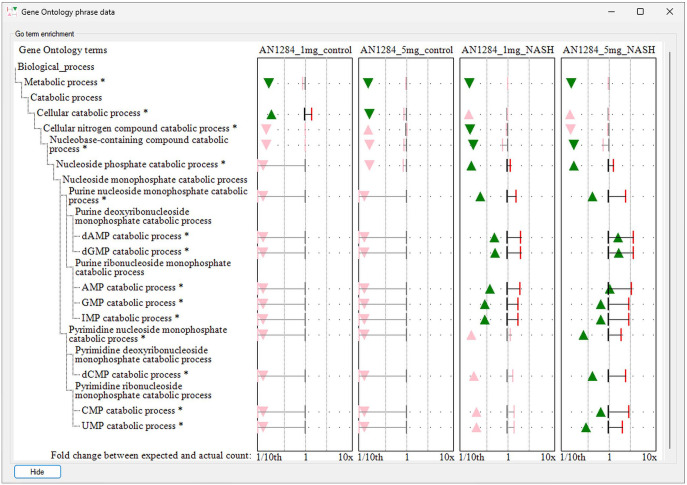
The secondary window of GOTermViewer visualizing the GO term enrichment for various terms linked to nucleoside monophosphate catabolism for a series of 4 differential expression analysis. Statistically significant enrichment of GO terms is shown by a green triangle whose position indicates the enrichment’s odds ratio and a red vertical bar that indicates the fold change of genes linked to a term compared to the expected number linked to the term. Triangles that point upwards indicate GO terms with more genes linked to them than expected, while triangles that point down have fewer than expected genes linked to them. While the figure displays the x-axis for fold change enrichment, it is possible to exchange this with the scale for the odds ratio which is in the range 0 to 25.

Once selected, a term’s data are displayed in the secondary window: this display consists of 2 areas, to the left the GO term’s name and its relationship to other terms are displayed, while to the right, the enrichment data are displayed with the results of multiple analyses shown as a series of graphs allowing their easy comparison (Figure 3). GOstats enrichment data consist of the GO term’s odds ratio value and its statistical significance *P* value as well as the observed and expected number of DEG linked to the term. The value of the odds ratio is shown by the location of a triangle, which is green for significant enrichment or pink of non-significant terms. The orientation of the triangle indicates if the term is enriched (the triangle points up) or underrepresented (triangle points down). Since the number of DEG varies between different analyses, the number of expected and observed genes linked to a GO term are not directly comparable between analyses. Consequently, the fold change in enrichment for each term is displayed, with this value identified by a red vertical bar (significant enrichment) or a grey vertical bar (non-significant enrichment).

Once the final display of the data has been finalized, it’s possible to save the analysis as an image for inclusion in a publication or thesis. Furthermore, a specifically selected list of GO term paths can also be saved and re-imported to recreate the display for further analysis, or as a starting point for the analysis of new but related datasets.

## Discussion

A number of programmes have been developed to visualize GO term enrichment data; however, these applications tend to focus on the display of aggregated data from a single data set. The process by which data are aggregated and positioned in a display can be very sensitive to differences in the enrichment data or the parameters used to process it. While they can still be highly informative for the display of a single analysis, this approach leads to displays that may not be amenable for the comparison of enrichment data from a data series such as a time course or dose-response experiments as the results will contain both subtle and obvious differences in the enrichment data. Consequently, we chose to display the comparison of a series of enrichment analysis in a manner similar to a hierarchical graph, however, rather than displaying each data point as part of a hieratical graph, the order and indentation of a GO term’s name is used to display its relationship to other GO terms and then the linked data for each analysis is shown as a series of graphs to the right of the GO term’s name.

By allowing the user to select which GO terms are visualized and then displaying the selected GO terms in a highly detailed, unaggregated manner, it is possible to circumvent many of the problems associated with displaying GO term enrichment data as a bubble plot or network node. While using the indentation of the GO terms’ labels to show the relationship between terms, rather than using nodes in hierarchical graph, allows multiple enrichment analysis to be displayed without making the display cluttered or cramped.

An example of the ability of GOTermViewer to succinctly and clearly visualize data from a series of related enrichment analysis is shown in Figure 3, and while the ‘Nucleoside phosphate catabolic process’ term is enriched in NASH mice irrespective of the dose of AN1284,^30^ it is only with the higher dose that the terms linked to pyrimidine catabolic processes are enriched showing the drug has a stronger effect on purine catabolism than pyrimidine catabolism. Similarly, by displaying the fold-change and odds ratio for each term, one can observe that the higher dose appears to be linked to an increase in the number of genes linked to the drug in NASH mice. While not statically significant, there also appears to be an increase in the number of genes linked to pyrimidine catabolism at the lower dose of AN1284 which may prompt future work to determine the effect of intermediate doses of AN1284 on nucleoside catabolism. GOTermViewer allows these findings to be readily identified in a way that would not be possible if the data were displayed using bubble plots with aggregated clusters of Go terms or displays that have a limited ability to display secondary information such as hierarchical and network graphs.

While GOTermViewer simplifies the comparison of GO term enrichments from a series of related experiments, it should be noted that the results should be seen as a guide to future work rather than a definitive answer. In particular, RNA-seq is prone to batch effects whereby experiments and/or sequencing performed at different times can have noticeable differences in the detected gene expression. These differences may arise from uncontrolled environmental factors affecting the cells during the experiment or use of different batches of reagents used to prepare and sequence the samples.

## Conclusions

There are many applications designed to display GO term enrichment data, the vast majority of these programmes are primarily aimed at the display of a single analysis. However, as the ease by which differential gene expression analyses can be performed as increased, experiments are increasingly being performed that contain multiple pairwise analysis such as a series of time courses or treatment regimes. However, there has not been a satisfactory way to compare the resultant series of GO term enrichment analyses; consequently, we have developed GOTermViewer to undertake this increasingly important task.
